# Supplementary material for: Phylogenetic Analysis Reveals Source Attribution Patterns for Campylobacter spp. in Tennessee and Pennsylvania
Source: Microorganisms. 2021 Nov 5;9(11):2300. doi: 10.3390/microorganisms9112300 (PMC8625337; doi:10.3390/microorganisms9112300)
Supplement: Supplementary file 1 [file FS2.pdf › supplemental/FS2.pdf]

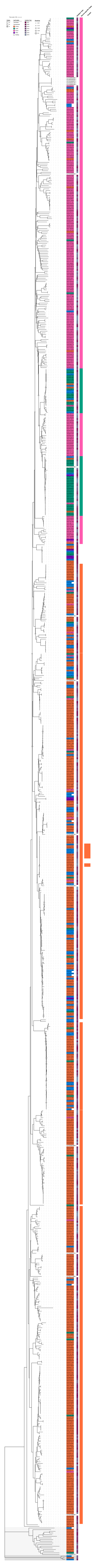

Tree scale: 100
Clades
1a
1b
1c
Leaf Labels
Tennessee
Pennsylvania
Poultry
Cattle
Swine
Other
RefSeq
Isolation Year
2021
2020
2019
2018
2017
2016
2015
Bootstrap
0.01
0.13
0.26
0.38
0.5
Isolation Year
Source-specific Clade
Cluster
