# Supplementary material for: Phylogenetic Analysis Reveals Source Attribution Patterns for Campylobacter spp. in Tennessee and Pennsylvania
Source: Microorganisms. 2021 Nov 5;9(11):2300. doi: 10.3390/microorganisms9112300 (PMC8625337; doi:10.3390/microorganisms9112300)
Supplement: Supplementary file 1 [file FS1.pdf › supplemental/FS1.pdf]

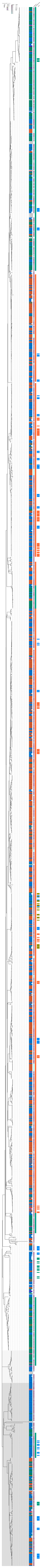

Tree scale: 100
Clades
1
2
3
Leaf Labels
Tennessee
Pennsylvania
Poultry
Cattle
Swine
Other
RefSeq
Isolation Year
2021
2020
2019
2018
2017
2016
2015
2014
2013
2003
2002
Bootstrap
0
0.13
0.25
0.38
0.5
Isolation Year
Source-Specific Clade
Cluster
